# hsa-miR-4443 inhibits myocardial fibroblast proliferation by targeting THBS1 to regulate TGF-β1/α-SMA/collagen signaling in atrial fibrillation

**DOI:** 10.1590/1414-431X202010692

**Published:** 2021-03-03

**Authors:** Jingwen Xiao, Yan Zhang, Yuan Tang, Hengfen Dai, Yu OuYang, Chuanchuan Li, Meiqin Yu

**Affiliations:** 1 Department of Cardiovascular Medicine, FuZhou First Hospital, FuZhou, Fujian, China; 2 Cardiac Function Laboratory of Cardiovascular Medicine, FuZhou First Hospital, FuZhou, Fujian, China; 3 Department of Clinical Pharmacy, FuZhou First Hospital, FuZhou, Fujian, China

**Keywords:** Atrial fibrillation, hsa-miR-4443, THBS1, TGF-β1/α-SMA/collagen pathway, Cardiac fibroblasts, Proliferation

## Abstract

Fibrosis caused by the increase in extracellular matrix in cardiac fibroblasts plays an important role in the occurrence and development of atrial fibrillation (AF). The aim of this study was to investigate the role of hsa-miR-4443 in AF, human cardiac fibroblast (HCFB) proliferation, and extracellular matrix remodeling. TaqMan Stem-loop miRNA assay was used to measure hsa-miR-4443 expression in patients with persistent AF (n=123) and healthy controls (n=100). Patients with AF were confirmed to have atrial fibrosis by late gadolinium enhancement. At the cellular level, after hsa-miR-4443 mimic and inhibitor were transfected with HCFBs, proliferation, apoptosis, migration, and invasion were analyzed. Lastly, hsa-miR-4443-targeted gene and transforming growth factor (TGF)-β1/α-SMA/collagen pathway were evaluated by dual-luciferase reporter assay and western blot, respectively. In patients with AF, hsa-miR-4443 decreased significantly and collagen metabolism level increased significantly. Logistic regression analysis showed that low hsa-miR-4443 level was a risk factor of AF (P<0.001). The receiver operating characteristic curve revealed that hsa-miR-4443 was useful for predicting AF (area under the curve: 0.828, sensitivity: 0.71, specificity: 0.78, P<0.001). In HCFBs, hsa-miR-4443 targeted thrombospondin-1 (THBS1) and downregulated TGF-β1/α-SMA/collagen pathway. The inhibition of hsa-miR-4443 expression promoted HCFB proliferation, migration, invasion, myofibroblast differentiation, and collagen production. The significant reduction of hsa-miR-4443 can be used as a biomarker for AF. hsa-miR-4443 protected AF by targeting THBS1 and regulated TGF-β1/α-SMA/collagen pathway to inhibit HCFB proliferation and collagen synthesis.

## Introduction

Atrial fibrillation (AF) is the most common arrhythmia in clinical practice, predominantly related to valvular, hypertension, or ischemic disease and it substantially enhances the possibility of stroke and heart failure (1,2). Atrial fibrosis is a major clinical marker of AF and an important step in the development of arrhythmia (3,4). Human cardiac fibroblasts (HCFBs) are the most abundant cell types in the heart. The core functions of HCFBs are to maintain the homeostasis of the cardiac extracellular matrix (ECM) and provide structural and mechanical support to the myocytes (5). However, pathological stimuli, such as myocardial injury, oxidative stress, mechanical traction, and inflammation, could promote HCFB proliferation, migration, and differentiation, thereby leading to ECM remodeling. Myofibroblasts differentiated from HCFBs would secrete a large amount of type I and type III collagen fibers and release growth factors, cytokines, and chemokines to maintain HCFB proliferation and atrial fibrosis (5). The fibrosis of the cardiac atrium will break the homogeneity of the atrial structure, increase its anisotropy, cause abnormal atrial electrophysiology, and generate and maintain AF (6). The intervention of atrial fibrosis is an effective method to prevent AF (7).

Currently, the following three main pathways that regulate the progression of atrial fibrosis are the renin-angiotensin-aldosterone system (8), the transforming growth factor (TGF)-β/Smad pathway (9,10), and the oxidative stress pathways (11). Among these pathways, TGF-β is a key effector of atrial fibrosis (12,13). The TGF-β1, N-terminal type I procollagen propeptide (PINP), carboxyl-terminal peptide from procollagen I (PICP), and N-terminal type III procollagen propeptide (PIIINP) levels significantly increase in patients with persistent AF, which suggests that types I and III collagen synthesis increase (14). The activation of the TGF-β1/α-SMA/collagen I profibrotic pathway contributes actively to atrial fibrosis in AF experimental model and patients (15). TGF-β1 induces α-SMA expression, and α-SMA is a sign of fibroblast activation and differentiation into myofibroblast (16). In a mouse model, the overexpression of TGF-β induces atrial interstitial fibrosis and AF vulnerability (10,17). Similar results were also obtained in lung fibrosis and liver fibrosis experiments (18,19). Therefore, TGF-β is an important target for AF treatment.

MicroRNAs (miRNAs) have physiological and pathophysiological functions in regulating cardiac fibrosis. For example, miR-128-3p, miR-495, miR-675, and miR-30e target Sp1, NOD1, TGFβR1, and Snai1, respectively, to inhibit TGF-β/Smad signal, thereby reducing HCFB proliferation and ECM accumulation to protect the heart from fibrosis (20–23). miR-216a is significantly upregulated in heart failure and activates Akt/mTOR and TGF-βRI/Smad2 signals by decreasing PTEN and Smad7 expression levels, thereby promoting HCFB proliferation and enhancing cardiac fibrosis (24). hsa-miR-96 is significantly upregulated in patients with AF and positively promotes HCFB proliferation, migration, and collagen I/III production (25). A large number of miRNAs affect atrial fibrosis by regulating HCFBs activity. In this study, hsa-miR-4443 in patients with AF was significantly reduced and negatively correlated with PINP, PICP, PIIINP, and left atrial diameter (LAD). LAD is also related to morbidity and relapse of atrial fibrosis (26). Analysis through TargetScan and the database for Annotation, Visualization, and Integrated Discovery (DAVID) showed that hsa-miR-4443 targets thrombospondin-1 (THBS1) to regulate the TGF-β signal. Therefore, we hypothesized that hsa-miR-4443 regulates atrial fibrosis and affects AF. Herein, we studied the role of hsa-miR-4443 in HCFB-to-myofibroblast differentiation and fibrosis, which are the two major features involved in cardiac remodeling, and explored their potential mechanisms.

## Material and Methods

### Patients

One hundred and twenty-three patients with persistent AF (continuous AF for at least 6 month) (14) at the Fuzhou First Hospital between January 2017 and May 2020 were included in the study. The late gadolinium enhancement cardiovascular magnetic resonance image was used to indicate ventricular fibrosis (27). The exclusion criteria were as follows: i) structural heart disease; ii) severe peripheral atherosclerotic disease; iii) history of some diseases (e.g., hyperthyroidism) that influence AF risk; iv) renal dysfunction, type II diabetes, acute or chronic infection, inflammatory disease, or fibrosis disease; and v) ongoing treatment with antiarrhythmics, lipid-lowering medication, and steroidal or nonsteroidal anti-inflammatory drugs. All patients underwent physical examination, routine laboratory tests, and 12-lead electrocardiogram or Holter test. Left ventricular ejection fraction (LVEF) was measured by ultrasonography within 24 h according to Simpson's biplane method (28), and LAD was calculated by transthoracic echocardiography. A total of 100 healthy volunteers with no clinical and electrocardiograph evidence of AF and no structural heart disease were randomly selected. All exclusion criteria with regard to comorbidities that were applied to the AF groups were also applied to the controls. Information on demographic and clinical characteristics was obtained for all recruited patients. The Ethics Committee of Fuzhou First Hospital approved the study protocol, and all patients provided signed informed consent forms. The investigation adhered to the principles outlined in the Declaration of Helsinki.

GE Vivi d95 color Doppler ultrasound system (GE, USA) and M5Sc transthoracic 2D phased array probe (3.5 MHz) were used for the ultrasound examination of all patients. Patients were in left lateral position, connected to the electrocardiogram, and the LAD was measured and recorded on the long axis of the left ventricle. The left ventricular end-systolic volume (LVESV), left ventricular end-diastolic volume (LVEDV), and left atrial volume were measured using the modified Simpson's biplane method. To calculate the LVEF, we used the following formula: LVEF = (LVEDV − LVESV) / LVEDV × 100%.

### Sample collection

Samples were collected from the patient's cubital vein using Monovette Sarstedt tubes (Sarstedt, Germany). Venous blood samples from each patient were collected by blood collection tubes. Serum was separated by centrifugation at 1800 *g* for 5 min at room temperature, and stored at −80°C until analysis. Given that hemolysis can cause a false increase either in common laboratory parameters or miRNA levels, visually detected hemolyzed serum samples were rejected.

### RNA extraction and quantitative real time-polymerase chain reaction (qRT-PCR)

Serum samples from patients and healthy donors were stored at −80°C until examination. After they were thawed, the samples were centrifuged at 3000 *g* for 5 min to pellet any debris and insoluble component. Circulating miRNA was extracted using the miRCURY™ RNA Isolation Kit (Exiqon, Denmark). For normalization, 100 ng exogenous and synthetic *Caenorhabditis elegans* miR-39 was added to each sample before RNA extraction. Purified miRNA was eluted with 20 µL of RNase-free water. The quantity and quality of the isolated miRNA were assessed using Agilent Bioanalyzer 2100 (Small RNA Analysis Chip, Agilent Technologies, Inc., USA). Reverse transcription was performed using the TaqMan miRNA Reverse Transcription kit (Applied Biosystems, USA). The reaction conditions were 16°C for 30 min, 42°C for 30 min, and 85°C for 5 min, followed by maintaining the temperature at −20°C. Then, 10 µL of TaqMan^®^ Universal PCR Master Mix (Applied Biosystems), 1 µL of TaqMan Stem-loop miRNAassay, 7.5 µL of nuclease free water, and 1.5 µL of RT product were used for real-time PCR reaction. Forty cycles of PCR amplification were performed, with initial incubation at 95°C for 10 min. Each cycle was done at 95°C for 15 s and annealing at 60°C for 1 min. The stem-loop primers were 5 nM each: cel-miR-39: GTCGTATCCAGTGCAGGGTCCGAG-GTATTCGCACTGGATACACCAAGCT; has-miR-4443: GTCGTATCCAGTGCAGGGTCCGAGGTATTCGCACTGGATACGACAAAACC.

The total RNA in HCFBs was isolated and treated with RNase-free water according to the TRIzol^®^ (Invitrogen, USA) method. Reverse transcription was performed using the Reverse Transcription System kit (Takara, China). The reaction conditions were 42°C for 60 min, followed by cooling to 4°C. The resultant complementary DNA (cDNA) was used as a template for subsequent PCR. Then, the specific primers were resuspended by adding 250 µL of RNase-free water. Each single real-time PCR reaction included 10 µL of qPCR Universal SYBR^®^ Green Master Mix, 10 µL of the primer set, 3 µL of RNase free water, and 2 µL of the RT product. Forty cycles of PCR amplification were performed, with initial incubation at 95°C for 10 min and final extension at 72°C for 5 min. Each cycle comprised denaturation at 95°C for 10 s, annealing at 60°C for 30 s, and extension at 72°C for 30 s. The reaction included a specific primer set and control primers for candidate genes: THBS1-F: 5′-GGCACCAACCGCATTCCAGAG-3′, THBS1-R: 5′-GCACAGCATCCACCAGGTCTTG-3′; GAPDH-F: 5′-GTCAAGGCTGAGAACGGGAA-3′, GAPDH-R: 5′-AAATGAGCCCCAGCCTTCTC-3′.

The mRNA expression level of hsa-miR-4443 was normalized to *C. elegans* miR-39, and the mRNA expression level of THBS1 was normalized to GAPDH. The relative expression of the candidate genes were calculated using the formula ΔΔCt = (Ct_sample_ - Ct_internal reference_) AF − (Ct_sample_ − Ct_internal reference_) control, and the estimated expression ratio was equal to 2^-ΔΔCt^.

### Enzyme-linked immunosorbent serologic assay (ELISA)

Serum PIIINP, PINP, and PICP levels were determined by sensitive ELISA kits (Lianshuo Biological Technology Co., Ltd., China) according to the manufacturer's instructions. Assays were performed in duplicate in a single run and normalized to a standard curve.

### Cell culture and transfection

Normal HCFBs were purchased and cultured according to the supplier's protocol (CC-2903, Lonza, Switzerland). Specifically, HCFBs were seeded in 24-well plates, each well containing 1×10^5^ cells. HCFBs were cultured in 10% FBS DMEM-F12 (Gibco, USA) in a humidified atmosphere of 5% CO_2_ at 37°C for 24 h. Lipofectamine 2000 Transfection reagent (Invitrogen) was utilized for cell transfection. hsa-miR-4443 mimic and inhibitor (GenePharma, China) were used to achieve ectopic miRNA expression. When HCFBs grew to 30-50% confluence, hsa-miR-4443 mimic (10 nM), inhibitor (10 nM), and negative control were utilized to transfect the cells for 48 h. Cells were collected and examined for overexpression or knockdown efficiency.

### CCK-8 assay for cell proliferation

We utilized a CCK-8 assay to measure HCFB proliferation. A total of 4×10^3^ cells/well were cultured in 96-well plates. miR-4443 mimic, inhibitor, and negative control were used to transfect cells. Then, all wells were treated with 100 µL of 10% CCK-8 reagent at 0, 24, 48, 72, and 96 h and cultured at 37°C for an additional 1-2 h. The absorbance value was measured at a 450 nm wavelength with a full wavelength microplate analyzer (Molecular Device, USA).

### Flow cytometric analysis

A total of 1×10^5^ cells/well were seeded in 24-well plates and transfected after 24 h. At 24 h post-transfection, the treated HCFBs were collected and washed two times with cold phosphate saline buffer. The cells were resuspended with binding buffer, and the concentration was adjusted to 1×10^6^ cells/mL. Then, 100 µL of HCFB suspension, 5 μL of annexin-FITC, and 10 μL (20 μg/mL) of propidium iodide were added to the flow tube and subsequently incubated in the dark. Finally, apoptosis was determined by flow cytometry analysis.

### Cell transwell assays for migration and invasion

The migration and invasion capabilities of HCFBs were examined by transwell assays. A total of 1×10^5^ transfected cells/well were seeded in the transwell top chamber of an insert with serum-free media for the migration assay. The transwell top chamber was coated with 25 mg Matrigel, and then 1×10^5^ cells/well were added to the chamber for the invasion assay. The cells on the top of the membrane were removed with cotton swabs after 24 h. The invaded or migrated cells on the bottom chamber were fixed by 4% paraformaldehyde for 30 min and stained by 0.1% crystal violet for an additional 15 min. The numbers of invaded and migrated HCFBs were calculated in six randomly chosen microscopic fields (200×) per membrane. The following formula was used: relative migration/invasion cells (%) = the number of cells in experimental group / control group × 100%. All assays were performed independently in triplicate.

### Luciferase reporter assay

THBS1 3′-UTR was amplified from the full-length cDNA obtained from Open Biosystems (NCBI, USA) via PCR using the primers: THBS1-F: 5′- CCTCGAGACCAATGCTGGTATTGCACC-3′ and THBS1-R: 5′- ATTTGCGGCCGCATGGCCTCACAATAGCACCC-3′. Then, THBS1 3′-UTR was digested with XhoI and NotI, and the 3′-UTR fragment was inserted into the XhoI/NotI sites of the psiCHECK-2 vector (Promega, USA) to obtain a 3′-UTR-wild-type (WT) luciferase reporter plasmid. The THBS1 3′-UTR-mutant (Mut) was constructed (GenePharma) and inserted into the psiCHECK-2 vector.

Then, either THBS1 WT or Mut 3′-UTR reporter plasmids was co-transfected with miR-4443 mimic or miR-4443 mimic negative control into HCFBs using Lipofectamine 2000 Transfection reagent (Invitrogen). Specifically, 1×10^5^ of HCFBs/well were seeded in 24-well plates. When HCFBs grew to 70% confluence, cells were co-transfected with THBS1-3′-UTR reporter plasmid (50 ng) and 10 nM of miR-4443 mimic or negative control. After 48 h post-transfection, firefly and Renilla luciferase activities were detected according to the dual-luciferase reporter assay (Promega). The Renilla luciferase values were then divided by the firefly luciferase activity values to normalize the difference in transfection efficiency. The experiments were performed in triplicate.

### Western blot analysis

The extracted protein concentration was calculated using a BCA protein assay kit (Beyotime, China). A total of 20 μg of each protein sample were separated by 10% SDS-PAGE, transferred to a pure nitrocellulose membrane (BioTrace, USA), blocked with 5% BSA for 2 h, and subsequently incubated with primary antibodies specific for THBS1, TGF-β, Smad2/3, p-Smad2, p-Smad3, LTBP1, collagen I, collagen III, and α-SMA (1:1000, Cell Signaling Technology, USA), anti-β-actin (1:5000), and anti-GAPDH (1:5000) at 4°C overnight. The next day, membranes were further incubated with an appropriate anti-mouse antibody for an additional 1-2 h. Chemiluminescent detection was performed using an ECL western blot detection kit (Thermo, USA). Bands were imaged by Tanon 5200 Biotanon (China).

### Statistical analysis

All experiments were analyzed by SPSS 20.0 (IBM, USA) software. If data followed a normal distribution, Student's *t*-test was performed to compare the differences between two groups, and one-way ANOVA analysis with LSD *post hoc* test to compare the differences between more than two groups. If the data did not follow a normal distribution, Mann-Whitney U test was used. The correlation of serum hsa-miR-4443 concentrations with clinical parameters was analyzed by Spearman correlation analysis. Logistic regression analysis was used to assess the association of factors. The clinical accuracy of significant hsa-miR-4443 was assessed using receiver operator characteristic (ROC) analysis, and the area under the curve (AUC) was calculated by DeLong's method. P<0.05 was considered to be a significant difference.

## Results

### Baseline clinical characteristics

The clinical data for the AF and control groups are listed in Table 1. In brief, the study group consisted of 123 patients with persistent AF (55% men), and the average age was 61.35±0.82 years old. The control group included 100 non-AF patients (52% men), and the average age was 61.19±0.81 years old. The study group was characterized with a significantly high level of C-reactive protein (CRP), low-density lipoprotein cholesterol (LDL-C), high-density lipoprotein cholesterol (HDL-C), total cholesterol (TC), PINP, PICP, PIIINP, and LAD (P<0.05), but LVEF (P<0.001) was significantly decreased. The differences in age, sex, body mass index (BMI), and triglyceride (TG) between the two groups (P=0.630, P=0.625, P=0.089, and P=0.090, respectively) were not significant. These results suggested cardiac function reduction, fibrosis, and inflammatory responses in patients with AF.

**Table 1:** Clinical data for atrial fibrillation (AF) patients and control group.

Factors	AF (n=123)	Control (n=100)	P value
Age (years)	61.35±0.82	61.91±0.81	0.630^a^
Men	68 (55%)	52 (52%)	0.625^b^
BMI (kg/m^2^)	22.81±0.20	22.30±0.22	0.089^a^
CRP (mg/L)	5.60 (3.56-8.17)	1.81 (1.04-2.43)	<0.001^c^
LDL-C (mM)	2.94 (2.57-3.42)	1.69 (1.38-2.06)	0.024^c^
HDL-C (mM)	1.71±0.04	1.58±0.03	0.012^a^
TC (mM)	4.28±0.11	3.07±0.09	<0.001^a^
TG (mM)	1.78 (1.23-2.37)	1.56 (1.15-2.12)	0.090^c^
PINP (ng/mL)	41.00 (32.28-46.98)	9.03 (6.43-11.57)	<0.001^c^
PICP (ng/mL)	40.25 (30.47-46.26)	12.10 (8.51-14.96)	<0.001^c^
PIIINP (ng/mL)	71.36 (32.26-83.94)	15.20 (10.21-17.85)	<0.001^c^
LAD (mm)	44.30 (38.50-49.90)	35.75 (32.42-37.40)	<0.001^c^
LVEF (%)	58.60 (55.20-61.90)	64.05 (58.45-68.10)	<0.001^c^
Has-miR-4443	0.56 (0.29-0.83)	1.24 (0.72-2.27)	<0.001^c^

BMI: body mass index; CRP: C-reactive protein; LDL-C: low-density lipoprotein cholesterol; HDL-C: high-density lipoprotein cholesterol; TC: total cholesterol; TG: triglyceride; PINP: N-terminal type I procollagen propeptides; PICP: carboxyl-terminal peptide from pro-collagen I; PIIINP: N-terminal type III procollagen propeptides; LAD: left atrial diameter; LVEF: left ventricular ejection fraction. Data are reported as number and percentage, means±SD, and median with interquartile interval (Q1-Q3). ^a^Student's *t*-test; ^b^Chi-squared test; ^c^Mann-Whitney U test. P<0.05 was considered a significant difference.

### hsa-miR-4443 was downregulated in patients with AF

**Figure 1:**
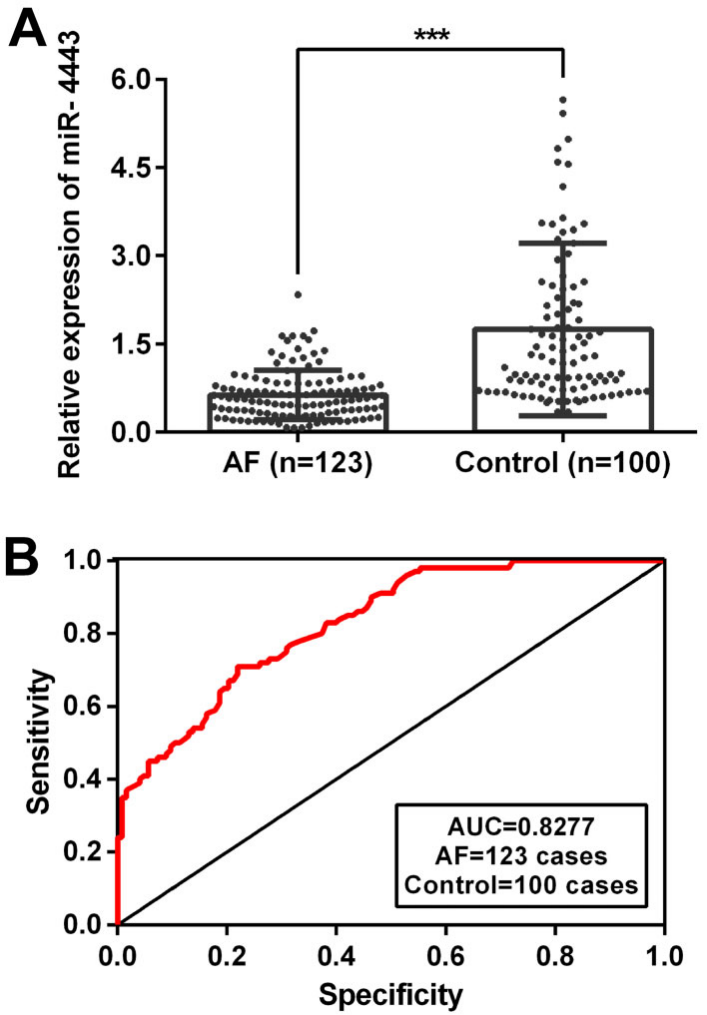
hsa-miR-4443 decreased in patients with atrial fibrillation (AF). **A**, Serum hsa-miR-4443 expression level was determined by qRT-PCR. **B**, ROC analysis was performed to evaluate the diagnostic efficacy of serum hsa-miR-4443 for persistent AF. Horizontal lines indicate median and interquartile range. ***P<0.001 *vs* control (Mann-Whitney U test, two-sided).

The mRNA level of hsa-miR-4443 was significantly lower in patients with AF than in control subjects (0.56 [0.29-0.83] *vs* 1.24 [0.72-2.27], P<0.001; Table 1 and Figure 1A). The decreased hsa-miR-4443 level may be associated with the pathogenesis of AF.

### Association of serum hsa-miR-4443 expression levels with clinical characteristics

According to Spearman's rank correlation, HDL-C (r [correlation coefficient]=-0.221, P=0.014), PINP (r=-0.383, P<0.001), PICP (r=-0.202, P=0.025), PIIINP (r=-0.181, P=0.045), and LAD (r=-0.290, P=0.001) had negative correlations with hsa-miR-4443 expression in the study group (Table 2). However, the expression of hsa-miR-4443 in the control group was not correlated with age, sex, BMI, CRP, LDL-C HDL-C, TC, TG, PINP, PICP, PIIINP, LAD, and LVEF (P>0.05). These results suggested that hsa-miR-4443 might regulate collagen metabolism in the pathogenesis of AF.

**Table 2:** Correlation between hsa-miR-4443 levels and clinical parameters in atrial fibrillation (AF) patients and control group.

Characteristic	AF		Control	
	r	P value	r	P value
Age (years)	0.027	0.763	-0.182	0.070
Men	0.132	0.146	-0.131	0.195
BMI (kg/m^2^)	0.098	0.298	-0.104	0.303
CRP (mg/L)	0.089	0.326	0.126	0.212
LDL-C (mM)	-0.118	0.195	0.060	0.554
HDL-C (mM)	-0.221	0.014	0.093	0.356
TC (mM)	-0.043	0.639	0.043	0.671
TG (mM)	-0.016	0.857	0.164	0.104
PINP (ng/mL)	-0.383	<0.001	0.082	0.415
PICP (ng/mL)	-0.202	0.025	-0.160	0.111
PIIINP (ng/mL)	-0.181	0.045	-0.070	0.487
LAD (mm)	-0.290	0.001	-0.026	0.798
LVEF (%)	0.130	0.151	-0.193	0.054

BMI: body mass index; CRP: C-reactive protein; LDL-C: low-density lipoprotein cholesterol; HDL-C: high-density lipoprotein cholesterol; TC: total cholesterol; TG: triglyceride; PINP: N-terminal type I procollagen propeptides; PICP: carboxyl-terminal peptide from pro-collagen I; PIIINP: N-terminal type III procollagen propeptides; LVEF: left ventricular ejection fraction; LAD: left atrial diameter; r: correlation coefficient. P<0.05 was considered a significant difference.

### Logistic regression analysis

The simple logistic regression analysis revealed that the following factors were risk factors for persistent AF: CRP (OR=0.273), LDL-C (OR=0.089), HDL-C (OR=0.431), TC (OR=0.366), PINP (OR=0.825), PICP (OR=0.835), PIIINP (OR=0.855), LAD (OR=0.734), LVEF (OR=1.214), and hsa-miR-4443 (OR=8.305) (Table 3).

**Table 3:** Logistic regression analysis of risk factors for atrial fibrillation.

Characteristic	Univariate logistic regression		
	OR	95%CI	P value
Age (years)	0.509	0.977-1.039	0.490
Men	0.670	0.672-1.938	0.338
BMI (kg/m^2^)	0.901	0.798-1.016	0.090
CRP (mg/L)	0.273	0.186-0.402	<0.001
LDL-C (mM)	0.089	0.048-0.164	<0.001
HDL-C (mM)	0.431	0.217-0.855	0.016
TC (mM)	0.366	0.270-0.498	<0.001
TG (mM)	0.679	0.435-1.059	0.088
PINP (ng/mL)	0.825	0.785-0.867	<0.001
PICP (ng/mL)	0.835	0.795-0.876	<0.001
PIIINP (ng/mL)	0.855	0.803-0.911	<0.001
LAD (mm)	0.734	0.675-0.799	<0.001
LVEF (%)	1.214	1.140-1.292	<0.001
hsa-miR-4443	8.305	4.205-16.405	<0.001

BMI: body mass index; CRP: C-reactive protein; LDL-C: low-density lipoprotein cholesterol; HDL-C: high-density lipoprotein cholesterol; TC: total cholesterol; TG: triglyceride; PINP: N-terminal type I procollagen propeptides; PICP: carboxyl-terminal peptide from pro-collagen I; PIIINP: N-terminal type III procollagen propeptides; LVEF: left ventricular ejection fraction; LAD: left atrial diameter; OR: odds ratio; 95%CI: 95% confidence intervals. P<0.05 was considered a significant correlation.

### ROC analysis

The result of ROC analysis revealed that the miR-4443 gradient <0.845 (AUC 0.828, 95%CI: 0.776 to 0.879, sensitivity: 0.71, specificity: 0.78, P<0.001) was a predictor for the persistent AF (Figure 1B).

### Effects of hsa-miR-4443 on cell proliferation and apoptosis

**Figure 2:**
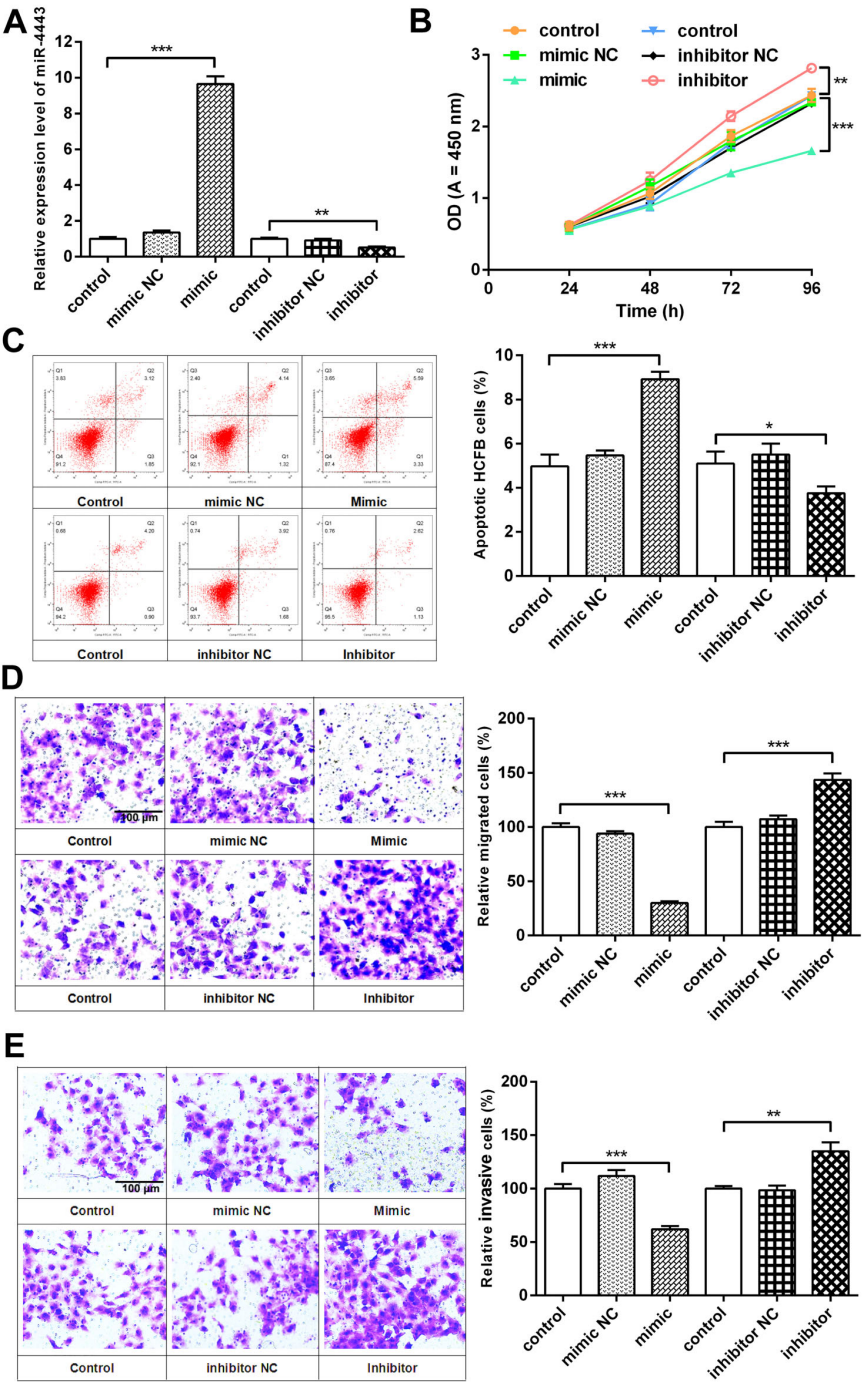
hsa-miR-4443 affected the biological function of human cardiac fibroblast (HCFBs). **A**, HCFBs were transfected with hsa-miR-4443 mimic or inhibitor. hsa-miR-4443 expression affected cell proliferation (**B**), apoptosis (**C**), migration (**D**), and invasion (**E**) (scale bars, 100 μm). All data are reported as means±SD of at least three independent experiments. *P<0.05, **P<0.01, and ***P<0.001 *vs* control (one-way ANOVA analysis with two-sided LSD *post hoc* test). NC: negative control.

miR-4443 expression was significantly increased in the mimic group (P<0.001) *vs* the control or negative control groups, and it was significantly decreased in the inhibitor group (P<0.01, Figure 2A). HCFBs were in a relatively static state at the physiological level and mainly played a supporting role in the heart. However, the proliferation of HCFBs decreased significantly in the miR-4443 mimic group, while the inhibitor promoted cell proliferation (Figure 2B). miR-4443 mimic also significantly increased the cell apoptosis rate, and the inhibitor decreased cell apoptosis (Figure 2C). Therefore, miR-4443 negatively controlled HCFB proliferation and promoted apoptosis. Reduced miR-4443 in AF patients might promote HCFB proliferation and inhibit apoptosis.

### Effects of hsa-miR-4443 on cell migration and invasion

miR-4443 mimic drastically suppressed HCFB migration (Figure 2D) and invasion (Figure 2E). However, transfection with miR-4443 inhibitor enhanced HCFB migration and invasion. Thus, the results indicated that miR-4443 knockdown contributed to the migration and invasion capacity of HCFBs, respectively.

### THBS1 was a target gene of hsa-miR-4443

**Figure 3:**
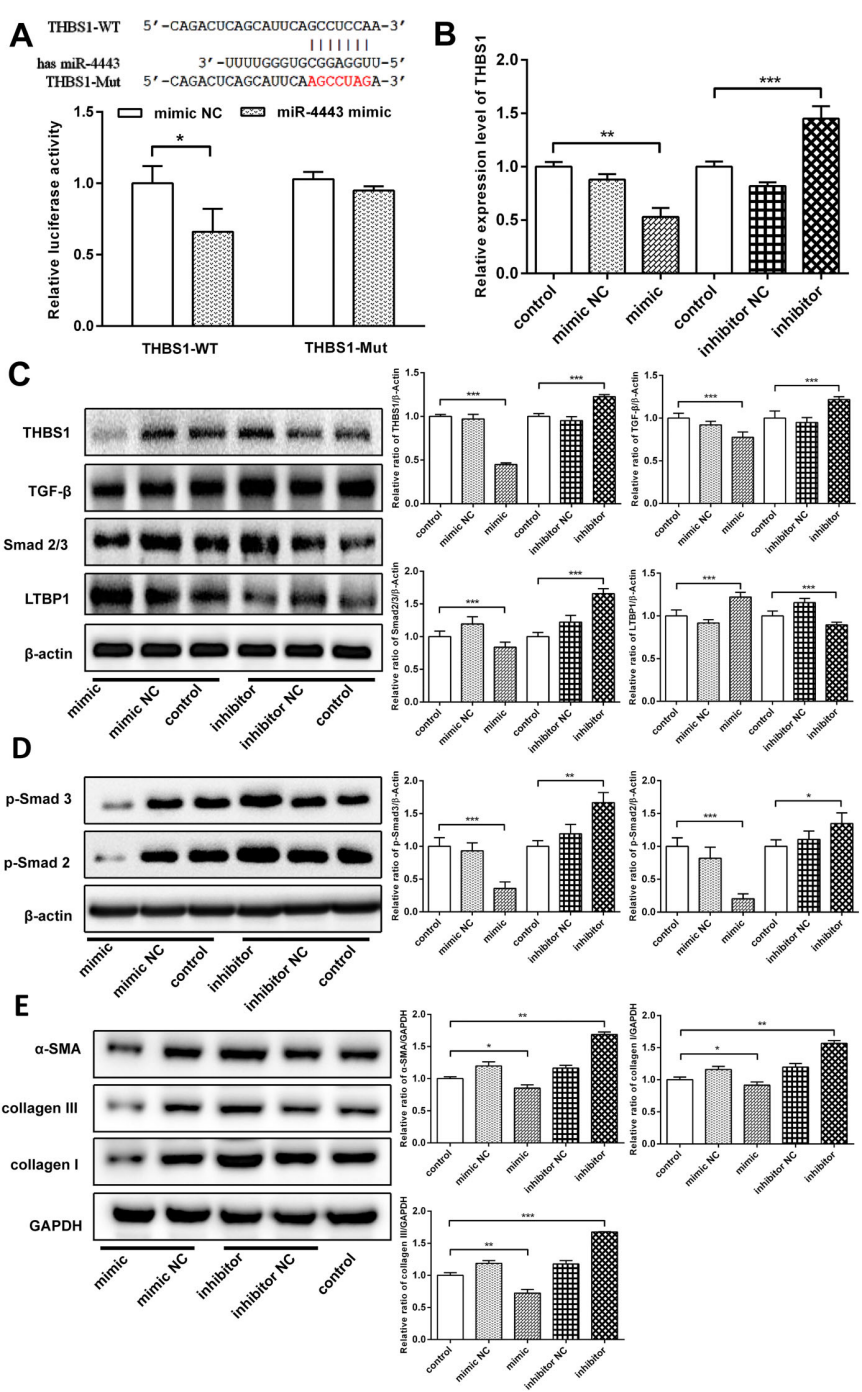
hsa-miR-4443 targeted THBS1 to regulate transforming growth factor (TGF)-β1/α-SMA/collagen signaling in human cardiac fibroblast (HCFBs). **A**, Binding sequences between hsa-miR-4443 and THBS1 3′-UTR transcripts, and dual-luciferase activity assay revealed hsa-miR-4443 targeted THBS1. **B**, hsa-miR-4443 promoted THBS1 mRNA degradation. **C**, The expression of THBS1, TGF-β, Smad2/3, and LTBP1 protein was examined in HCFBs. **D**, p-Smad2 and p-Smad3 expression levels were detected. **E**, α-SMA, collagen I, and collagen III protein expression levels were compared with the control group. All data are reported as means±SD of at least three independent experiments. *P<0.05, **P<0.01, and ***P<0.001 *vs* control (Student's *t*-test or one-way ANOVA analysis with two-sided LSD *post hoc* test). NC: negative control.

Analysis of the TargetScan databases showed that THBS1 may be the target gene of miR-4443 because a binding site for THBS1′-UTR was found in miR-4443 (Figure 3A). THBS1-3′-UTR-WT and 3′-UTR-Mut were then constructed and inserted into the psiCHECKTM-2 vector to structure reporter plasmid. To verify this prediction, the dual-luciferase reporter assay revealed that miR-4443 mimic transfection caused a significant reduction in the activity of THBS1-3′-UTR-WT but not of THBS1-3′-UTR- Mut (Figure 3A). The expression levels of THBS1 mRNA were measured by qRT-PCR in the miR-4443 mimic- and inhibitor-transfected HCFBs. THBS1 mRNA increased significantly in the miR-4443 inhibitor group. Conversely, the overexpression of miR-4443 significantly decreased THBS1 mRNA expression (P<0.001, Figure 3B). These results suggested that THBS1 was directly targeted by miR-4443 in HCFBs.

### hsa-miR-4443 suppressed TGF-β1/α-SMA/collagen signaling by directly targeting THBS1

To determine the molecular mechanism on how miR-4443/THBS1 functions as a mediator in HCFB proliferation, migration, and invasion, we analyzed the DAVID and KEGG databases and found THBS1 was the linkage of TGF-β1/α-SMA/collagen signaling, thereby regulating fibrosis. THBS1, TGF-β, Smad2/3, p-Smad2, and p-Smad3 protein significantly decreased in miR-4443 mimic-transfected cells compared with the control or negative control groups, and LTBP1 significantly increased (P<0.001). Conversely, the knockdown of miR-4443 remarkably enhanced the expression levels of THBS1, TGF-β, Smad2/3, p-Smad2, and p-Smad3 and significantly decreased LTBP1 expression (P<0.05, (Figure 3C and D). This result suggested that miR-444 regulated HCFB biological functions by targeting to suppress THBS1 expression and regulating TGF-β1/Smad signals. Then, miR-4443 upregulation inhibited α-SMA, collagen I, and collagen III expression (P<0.05), and miR-4443 downregulation increased α-SMA, collagen I, and collagen III expression (P<0.01, Figure 3E). This result indicated that miR-4443 regulated the phenotypic conversion of HCFBs to myofibroblasts and affected the synthesis of ECM.

## Discussion

In this study, hsa-miR-4443 was downregulated in patients with AF and had high sensitivity and specificity for AF diagnosis. The inhibition of hsa-miR-4443 expression promoted HCFB proliferation, migration, invasion, myofibroblast differentiation, and collagen production. The mechanism was through downregulation of hsa-miR-4443, which increased THBS1 expression to activate the TGF-β/Smad signal. This result indicated that hsa-miR-4443 can be used to predict AF.

In this study, hsa-miR-4443 significantly decreased in patients with AF compared with control and demonstrated that serum hsa-miR-4443 was a promising diagnostic and molecular biomarker. At present, no other research has been conducted on the role of miR-4443 in AF; our research group is the first one to study this topic. Downregulated hsa-miR-4443 in ovarian cancer contributes to metastasis and tumorigenesis (27). However, several studies revealed that hsa-miR-4443 is up-regulated in diseases such as acute ischemic stroke (28), central nervous system tumors (29), and drug-resistant non-small cell lung cancer (30), which leads to pathogenic processes. hsa-miR-4443 is detected in different samples, such as tissues, serum, monocytes, or exosomes, thereby leading to no comparability of hsa-miR-4443 expression among different diseases (27–30).

In the present study, Spearman correlation analysis showed that PINP, PICP, PIIINP, and LAD were negatively correlated with serum hsa-miR-4443 levels. PINP, PICP, and PIIINP were the serum markers of types I and III collagen synthesis, respectively. Increased serum PICP and PIIINP levels are positively correlated with left atrial fibrosis and AF development, respectively (31). The accumulation of types I and III collagen in the extracellular space prolongs the conduction time between muscle cells, thereby leading to arrhythmia. The size and heterogeneous spatial distribution of fibrotic patches govern AF dynamics and fractionation (32).

LAD is an independent predictor of AF recurrence after ablation (33). LAD size increases, thereby decreasing the left ventricular systolic function, increasing the atrial activation time, and inducing AF (34). Herein, the decrease in hsa-miR-4443 was a risk factor for AF and negatively correlated with PINP, PICP, PIIINP, and LAD. This result suggested that the decrease in hsa-miR-4443 may promote the synthesis of types I and III collagen, which decreased the left ventricular systolic function in AF.

Afterward, we investigated the role of miR-4443 in HCFBs. Overexpressed miR-4443 inhibited HCFB proliferation, migration, and invasion. According to reports, miR-4443 upregulation inhibits tumor cellular growth, migration, and invasion, and induces apoptosis (35,36). The underlying mechanisms are related to the activation of JAK2/STAT3 pathway, anti-inflammatory cytokines, matrix metalloproteinases, and epithelial-mesenchymal transition (28,30,35,36). However, another role for miR-4443 was found in the present study. The result of luciferase reporter gene indicated that THBS1 was a target of miR-4443. THBS1 is an extracellular matrix glycoprotein that mediates cell-matrix and cell-cell interactions (37). This protein activates TGF-β, which is a fibrosis and anti-inflammatory factor, and influences cardiac remodeling by affecting cardiomyocyte apoptosis, myofibroblast differentiation, collagen production, etc. miR-4443 that participates in myocardial fibrosis may be related to the regulation of THBS1/TGF-β signal. Therefore, we tested the expression of key node proteins in this pathway. First, miR-4443 knockdown increased THBS1, TGF-β, Smad2/3, p-Smad2, and p-Smad3 expression levels, and LTBP1 was significantly inhibited. This result indicated that miR-4443 negatively regulated THBS1/TGF-β signal. The restriction of LTBP1 would induce TGF-β activation and mediate downstream Smad phosphorylation/signaling (38). TGF-β1 overexpression induces cardiac fibrosis, which alters the myocardial structure to produce a substrate for AF, such as increased heterogeneity of atrial conduction and conduction velocity (10,13).

The activation of TGF-β/Smad signal mainly induces cardiac fibrosis by increasing myofibroblast numbers and collagen deposition (12,15). Therefore, we further evaluated α-SMA and collagen expression. In this study, the α-SMA, collagen I, and collagen III expression levels were upregulated by miR-4443 inhibitor. α-SMA was a specific smooth muscle marker molecule. High α-SMA expression level suggested HCFBs transformed into myofibroblast phenotype. Myofibroblasts are the main cells to secrete ECM. The remodeling of ECM is the major manifestation of atrial fibrosis, and the main components of ECM are collagens I and III (39). Hence, miR-4443 expression could inhibit the HCFBs transformation into myofibroblasts and reduced collagen accumulation in the ECM. Here, miR-4443 acted as a modulator of THBS1 to regulate TGF-β1/α-SMA/collagen pathway and affected HCFB proliferation, migration, collagen production, and atrial fibrosis. miR-4443 may be a biomarker of AF.

In conclusion, this study revealed that hsa-miR-4443 is a key factor in the pathogenesis of AF (40), where it inhibited cell proliferation, migration, invasion, and myofibroblast phenotypic transformation in HCFBs while promoting apoptosis by directly regulating THBS1 expression and inactivating the downstream pathway TGF-β1/Smad. Therefore, increasing hsa-miR-4443 expression may be a potent strategy for THBS1 treatment. This study provided a novel understanding of the pathogenesis of AF and may provide a promising therapeutic approach for AF. However, the results of this study still need to be verified in a large number of clinical samples, further mechanism research, and animal models.

## References

[1] Nattel S, Harada M (2014). Atrial remodeling and atrial fibrillation: recent advances and translational perspectives. J Am Coll Cardiol.

[2] Chen LY, Shen WK (2007). Epidemiology of atrial fibrillation: a current perspective. Heart Rhythm.

[3] Nattel S (2017). Molecular and cellular mechanisms of atrial fibrosis in atrial fibrillation. JACC Clin Electrophysiol.

[4] Burstein B, Nattel S (2008). Atrial fibrosis: mechanisms and clinical relevance in atrial fibrillation. J Am Coll Cardiol.

[5] Jalife J, Kaur K (2015). Atrial remodeling, fibrosis, and atrial fibrillation. Trends Cardiovasc Med.

[6] Dzeshka MS, Lip GY, Snezhitskiy V, Shantsila E (2015). Cardiac fibrosis in patients with atrial fibrillation: mechanisms and clinical implications. J Am Coll Cardiol.

[7] Everett TH 4th, Olgin JE (2007). Atrial fibrosis and the mechanisms of atrial fibrillation. Heart Rhythm.

[8] Xiao HD, Fuchs S, Campbell DJ, Lewis W, Dudley SC, Kasi VS (2004). Mice with cardiac-restricted angiotensin-converting enzyme (ACE) have atrial enlargement, cardiac arrhythmia, and sudden death. Am J Pathol.

[9] Lijnen PJ, Petrov VV, Fagard RH (2000). Induction of cardiac fibrosis by transforming growth factor-beta (1). Mol Genet Metab.

[10] Verheule S, Sato T, Everett T 4th, Engle SK, Otten D, Rubart-von der Lohe M (2004). Increased vulnerability to atrial fibrillation in transgenic mice with selective atrial fibrosis caused by overexpression of TGF-beta1. Circ Res.

[11] Mihm MJ, Yu F, Carnes CA, Reiser PJ, McCarthy PM, Van Wagoner DR (2001). Impaired myofibrillar energetics and oxidative injury during human atrial fibrillation. Circulation.

[12] Sun Y, Huang ZY, Wang ZH, Li CP, Meng XL, Zhang YJ (2015). TGF-β1 and TIMP-4 regulate atrial fibrosis in atrial fibrillation secondary to rheumatic heart disease. Mol Cell Biochem.

[13] Choi EK, Chang PC, Lee YS, Lin SF, Zhu W, Maruyama M (2012). Triggered firing and atrial fibrillation in transgenic mice with selective atrial fibrosis induced by overexpression of TGF-β1. Circ J.

[14] Zhao F, Zhang S, Chen Y, Gu W, Ni B, Shao Y (2014). Increased expression of NF-AT3 and NF-AT4 in the atria correlates with procollagen I carboxyl terminal peptide and TGF-β1 levels in serum of patients with atrial fibrillation. BMC Cardiovasc Disord.

[15] Shen H, Wang J, Min J, Xi W, Gao Y, Yin L (2018). Activation of TGF-β1/α-SMA/ Col I profibrotic pathway in fibroblasts by Galectin-3 contributes to atrial fibrosis in experimental models and patients. Cell Physiol Biochem.

[16] Davis J, Molkentin JD (2014). Myofibroblasts: trust your heart and let fate decide. J Mol Cell Cardiol.

[17] Lee KW, Everett TH 4th, Rahmutula D, Guerra JM, Wilson E, Ding C (2006). Pirfenidone prevents the development of a vulnerable substrate for atrial fibrillation in a canine model of heart failure. Circulation.

[18] Iyer SN, Gurujeyalakshmi G, Giri SN (1999). Effects of pirfenidone on transforming growth factor-beta gene expression at the transcriptional level in bleomycin hamster model of lung fibrosis. J Pharmacol Exp Ther.

[19] García L, Hernández I, Sandoval A, Salazar A, Garcia J, Vera J (2002). Pirfenidone effectively reverses experimental liver fibrosis. J Hepatol.

[20] Cao F, Li Z, Ding WM, Yan L, Zhao QY (2019). LncRNA PVT1 regulates atrial fibrosis via miR-128-3p-SP1-TGF-β1-Smad axis in atrial fibrillation. Mol Med.

[B21] Wang X, Jin H, Jiang S, Xu Y (2018). MicroRNA-495 inhibits the high glucose-induced inflammation, differentiation and extracellular matrix accumulation of cardiac fibroblasts through downregulation of NOD1. Cell Mol Biol Lett.

[B22] Wang L, Jiang P, He Y, Hu H, Guo Y, Liu X (2019). A novel mechanism of Smads/miR-675/TGFβR1 axis modulating the proliferation and remodeling of mouse cardiac fibroblasts. J Cell Physiol.

[23] Zhang W, Chang H, Zhang H, Zhang L (2017). MiR-30e attenuates isoproterenol-induced cardiac fibrosis through suppressing Snai1/TGF-β signaling. J Cardiovasc Pharmacol.

[24] Tao J, Wang J, Li C, Wang W, Yu H, Liu J (2019). MiR-216a accelerates proliferation and fibrogenesis via targeting PTEN and SMAD7 in human cardiac fibroblasts. Cardiovasc Diagn Ther.

[25] Su L, Yao Y, Song W (2020). Downregulation of miR-96 suppresses the profibrogenic functions of cardiac fibroblasts induced by angiotensin II and attenuates atrial fibrosis by upregulating KLF13. Hum Cell.

[26] Jin X, Pan J, Wu H, Xu D (2018). Are left ventricular ejection fraction and left atrial diameter related to atrial fibrillation recurrence after catheter ablation?: A meta-analysis. Medicine (Baltimore).

[27] Ebrahimi SO, Reiisi S (2019). Downregulation of miR-4443 and miR-5195-3p in ovarian cancer tissue contributes to metastasis and tumorigenesis. Arch Gynecol Obstet.

[28] Li S, Lu G, Wang D, He JL, Zuo L, Wang H (2020). MicroRNA-4443 regulates monocyte activation by targeting tumor necrosis factor receptor associated factor 4 in stroke-induced immunosuppression. Eur J Neurol.

[29] Drusco A, Fadda P, Nigita G, Fassan M, Bottoni A, Gardiman MP (2018). Circulating micrornas predict survival of patients with tumors of glial origin. EBioMedicine.

[30] Zhang W, Qiao B, Fan J (2018). Overexpression of miR-4443 promotes the resistance of non-small cell lung cancer cells to epirubicin by targeting INPP4A and regulating the activation of JAK2/STAT3 pathway. Pharmazie.

[31] Swartz MF, Fink GW, Sarwar MF, Hicks GL, Yu Y, Hu R (2012). Elevated pre-operative serum peptides for collagen I and III synthesis result in post-surgical atrial fibrillation. J Am Coll Cardiol.

[32] Tanaka K, Zlochiver S, Vikstrom KL, Yamazaki M, Moreno J, Klos M (2007). Spatial distribution of fibrosis governs fibrillation wave dynamics in the posterior left atrium during heart failure. Circ Res.

[33] Wu XY, Li SN, Wen SN, Nie JG, Deng WN, Bai R (2015). Plasma galectin-3 predicts clinical outcomes after catheter ablation in persistent atrial fibrillation patients without structural heart disease. Europace.

[34] Ruaengsri C, Schill MR, Lancaster TS, Khiabani AJ, Manghelli JL, Carter DI (2018). The hemodynamic and atrial electrophysiologic consequences of chronic left atrial volume overload in a controllable canine model. J Thorac Cardiovasc Surg.

[35] Wang J, Zhang Q, Wang D, Yang S, Zhou S, Xu H (2020). Microenvironment- induced TIMP2 loss by cancer-secreted exosomal miR-4443 promotes liver metastasis of breast cancer. J Cell Physiol.

[36] Gong J, Wang J, Liu T, Hu J, Zheng J (2018). lncRNA FEZF1-AS1 contributes to cell proliferation, migration and invasion by sponging miR-4443 in hepatocellular carcinoma. Mol Med Rep.

[37] Adams JC, Lawler J (2011). The thrombospondins. Cold Spring Harb Perspect Biol.

[38] Liu G, Cooley MA, Jarnicki AG, Borghuis T, Nair PM, Tjin G (2019). Fibulin-1c regulates transforming growth factor-β activation in pulmonary tissue fibrosis. JCI Insight.

[39] Li PF, He RH, Shi SB, Li R, Wang QT, Rao GT, Yang B (2019). Modulation of miR-10a-mediated TGF-β1/Smads signaling affects atrial fibrillation-induced cardiac fibrosis and cardiac fibroblast proliferation. Biosci Rep.

[40] Liu H, Qin H, Chen GX, Liang MY, Rong J, Yao JP, Wu ZK (2014). Comparative expression profiles of microRNA in left and right atrial appendages from patients with rheumatic mitral valve disease exhibiting sinus rhythm or atrial fibrillation. J Transl Med.

